# Impact of postoperative complications on clinical outcomes after gastrectomy for cancer: multicentre study

**DOI:** 10.1093/bjs/znaf043

**Published:** 2025-03-28

**Authors:** Sander J M van Hootegem, Margrietha van der Linde, Marcel A Schneider, Jeesun Kim, Felix Berlth, Yutaka Sugita, Peter P Grimminger, Gian Luca Baiocchi, Giovanni De Manzoni, Maria Bencivenga, Suzanne Gisbertz, Souya Nunobe, Han-Kwang Yang, Christian A Gutschow, Sjoerd M Lagarde, Hester F Lingsma, Bas P L Wijnhoven, the GastroBenchmark Consortium, Hidde Overtoom, Ines Gockel, René Thieme, Ewen A Griffiths, William Butterworth, Henrik Nienhüser, Beat Müller, Nerma Crnovrsanin, Felix Nickel, Suzanne S Gisbertz, Mark I van Berge Henegouwen, Philip H Pucher, Kashuf Khan, Asif Chaudry, Pranav H Patel, Manuel Pera, Mariagiulia Dal Cero, Carlos Garcia, Guillermo Martinez Salinas, Paulo Kassab, Osvaldo Antônio Prado Castro, Enrique Norero, Paul Wisniowski, Luke Randall Putnam, Pietro Maria Lombardi, Giovanni Ferrari, Rita Gudaityte, Almantas Maleckas, Leanne Prodehl, Antonio Castaldi, Michel Prudhomme, Simone Giacopuzzi, Riccardo Rosati, Francesco Puccetti, Domenico D’Ugo, Daniel Gero, Hyuk-Joon Lee, the GASTRODATA Consortium, Guillaume Piessen, Justine Lerooy, Johanna Wilhelmina van Sandick, Suzanne S Gisbertz, Mark I van Berge Henegouwen, Jessie Elliott, Paolo Morgagni, Arnulf H Hölscher, Martin Hemmerich, Stefan Mönig, Mickael Chevallay, Piotr Kołodziejczyk, Henk Hartgrink, Paulo Matos da Costa, Filipe Castro Borges, Andrew Davies, Cara Baker, William Allum, Sacheen Kumar, Wojciech Polkowski, Karol Rawicz-Pruszyński, Uberto Fumagalli Romario, Stefano De Pascale, Antonio Tarasconi, Daniel Reim, Ilaria Pergolini, Lucio Lara Santos, Pedro Carvalho Martins, Alberto Biondi, Riccardo Rosati, Maurizio Degiuli, Rossella Reddavid, Wojciech Kielan, Paul Magnus Schneider, Thomas Murphy

**Affiliations:** Department of Surgery, Erasmus Medical Centre, Rotterdam, The Netherlands; Department of Public Health, Erasmus Medical Centre, Rotterdam, The Netherlands; Department of Surgery & Transplantation, University Hospital Zürich, Zürich, Switzerland; Department of Surgery, Seoul National University Cancer Hospital, Seoul, Korea; Department of General, Visceral and Transplant Surgery, University Medical Centre Mainz, Mainz, Germany; Department of Surgery, University Hospital of Tuebingen, Tuebingen, Germany; Department of Gastroenterological Surgery, Cancer Institute Hospital of the Japanese Foundation for Cancer Research, Tokyo, Japan; Department of General, Visceral and Transplant Surgery, University Medical Centre Mainz, Mainz, Germany; Department of Surgery, University Hospital of Brescia, Brescia, Italy; Department of Surgery, University Hospital of Verona, Verona, Italy; Department of Surgery, University Hospital of Verona, Verona, Italy; Department of Surgery, Amsterdam UMC, University of Amsterdam, Amsterdam, The Netherlands; Cancer Centre Amsterdam, Cancer Treatment and Quality of Life, Amsterdam, The Netherlands; Department of Gastroenterological Surgery, Cancer Institute Hospital of the Japanese Foundation for Cancer Research, Tokyo, Japan; Department of Surgery, Seoul National University Cancer Hospital, Seoul, Korea; Department of Surgery & Transplantation, University Hospital Zürich, Zürich, Switzerland; Department of Surgery, Erasmus Medical Centre, Rotterdam, The Netherlands; Department of Public Health, Erasmus Medical Centre, Rotterdam, The Netherlands; Department of Surgery, Erasmus Medical Centre, Rotterdam, The Netherlands

## Abstract

**Background:**

To reduce the clinical and economic burden of complications after gastrectomy for gastric cancer, specific complications should be targeted to effectively allocate healthcare resources for quality improvement and preventive measures. The aim of this study was to assess the impact of complications on clinical outcomes.

**Methods:**

This was a retrospective multicentre study of patients who underwent (sub)total gastrectomy for gastric or junctional adenocarcinoma at 43 centres in 16 countries between 2017 and 2021. Outcomes were escalation of care, reoperation, prolonged hospital stay (greater than the 75th percentile), readmission, and 30-day mortality. Adjusted relative risks and population attributable fractions were estimated for specific complication–outcome pairs. The population attributable fraction represents the percentage reduction in the frequency of an adverse outcome if a complication could be completely prevented in the population.

**Results:**

In total, 7829 patients were included. Postoperative complications occurred in 1884 patients (24.1%). The most frequent complications were pulmonary complications (436 patients (5.6%)), anastomotic leakage (363 patients (4.6%)), and abdominal collection (301 patients (3.8%)). Anastomotic leakage, cardiac complications, and pulmonary complications had the greatest impact on 30-day mortality (population attributable fraction 26.6% (95% c.i. 14.5% to 38.6%), 18.7% (95% c.i. 9.4% to 28.0%), and 15.6% (95% c.i. 12.0% to 30.0%) respectively). Anastomotic leakage and pulmonary complications had the greatest impact on escalation of care (population attributable fraction 26.3% (95% c.i. 20.6% to 32.0%) and 18.4% (95% c.i. 11.7% to 25.2%) respectively), whereas anastomotic leakage and intra-abdominal bleeding had the greatest impact on reoperation (population attributable fraction 31.6% (95% c.i. 26.4% to 36.9%) and 8.5% (95% c.i. 5.5% to 11.5%) respectively). Most of the studied complications contributed to a prolonged hospital stay, whereas the contribution of complications to readmission did not exceed 15.9%. Subgroup analysis showed regional variation in the impact of complications.

**Conclusion:**

Anastomotic leakage had the largest overall negative impact on clinical outcomes after gastrectomy for gastric adenocarcinoma. Reducing the incidence of anastomotic leakage and pulmonary complications would have the most impact on the burden of complications.

## Teaser

This study assessed the impact of specific complications on clinical outcomes after gastrectomy for gastric cancer. It found that anastomotic leakage and pulmonary complications had the greatest negative impact on outcomes, including mortality, escalation of care, and reoperation, suggesting that targeting these complications could significantly reduce their overall burden.

## Introduction

The cornerstone of curative treatment for patients with gastric cancer is surgery, consisting of (sub)total gastrectomy plus lymphadenectomy. Although the implementation of minimally invasive procedures has minimized surgical trauma, gastrectomy still leads to postoperative complications in up to 20–40% of patients^1^. Consequently, there has been growing interest to monitor and compare outcomes for complex and cost-intensive surgical procedures^2–4^. With this aim, the GastroBenchmark Consortium and the GASTRODATA Consortium were conceived, accruing data from 43 centres. The recently published benchmark analysis^5^ provides reference data to compare outcomes after gastrectomy under ideal conditions, including clinical outcomes such as postoperative complications.

Early detection and effective treatment of complications are important to prevent adverse outcomes such as escalation of care, readmission, mortality, and diminished quality of life^6–10^. The downstream effects of these adverse outcomes also drive healthcare expenses, consume hospital resources, and result in lost productivity due to prolonged patient disability^10–12^. In this context, benchmarking provides a basis to improve care. However, further analysis is needed to prioritize complications that have the greatest clinical and economic impact. Targeted interventions for these complications may improve outcomes^13^.

On a population level, the impact of complications can be measured with the population attributable fraction (PAF), traditionally used in epidemiological literature^14^. This measure represents the burden of an outcome (for example mortality) that can be attributed to a specific risk factor (for example anastomotic leakage) by estimating the percentage reduction in the frequency of the outcome if the risk factor could be eliminated. The benefit of using the PAF is that it takes both frequency and relative risk (RR) into consideration, exposing the complications that have the most impact on a particular outcome.

In colorectal surgery, analyses by means of the PAF have led to a shift in targeted initiatives to improve outcomes^15–17^. For gastric cancer, a similar analysis was performed using a data set from the Netherlands^18^. However, the majority of gastric cancer patients are diagnosed in Asia, with different patient characteristics, treatment options, rates of postoperative complications, and resources. This complicates the understanding of the impact of complications due to differences in health care settings. The aim of this study was to assess the impact of complications on clinical outcomes after a gastrectomy for gastric cancer on a global scale and in a region-specific context to identify targets to reduce the clinical and economic burden of complications.

## Methods

### Database and ethics

This was a multicentre retrospective study of consecutive patients who underwent total and subtotal gastrectomy for gastric cancer between 1 January 2017 and 31 December 2021. Patients were included from the GastroBenchmark and GASTRODATA databases, containing data from 43 centres in 16 countries (*Supplementary material*); data were retrieved from these prospectively maintained databases and audited for completeness. Further details on data collection have been described previously^5^. The study committees of both the GastroBenchmark Consortium and the GASTRODATA Consortium approved the present study and provided the data for analysis. Before the start of this study, ethical approval was obtained from Erasmus MC (MEC-2023-0696) and, upon initiation of the database, approval was obtained from all participating centres.

### Patients and definition of complications

Patients with gastric adenocarcinoma who underwent elective (sub)total gastrectomy with curative intent were eligible for inclusion. Patients in whom no Roux-en-Y or Billroth reconstruction was created or no lymphadenectomy was performed were excluded. Complications were classified according to the format used in the KLASS trials for East Asian patients and the criteria published by the GASTRODATA group^19–21^. For centres using the GASTRODATA format, cardiac complications were only recorded when classified as major (requiring intervention or escalation of care as per GASTRODATA criteria) and pneumonia was recorded when a patient was symptomatic in combination with a positive culture. Other investigated complications were recorded irrespective of severity or method of diagnosis. Upon creation of the database, complications were appraised and grouped to have consistent definitions.

### Outcomes

The main outcomes were escalation of care (defined as unplanned readmission to a unit of higher surveillance), reoperation (surgical intervention under general anaesthesia), prolonged hospital stay (length of hospital stay greater than or equal to the 75th percentile, stratified for surgical access)^22^, readmission, and 30-day mortality.

### Statistical analysis

Multiple imputation with chained equations (5 iterations) was performed to impute missing data^23^. After assessment of possible correlation between outcomes and auxiliary variables, multiple imputation then deletion was performed to obtain the most accurate estimates of the missing values^24,25^. This means that if a patient had a missing outcome, they were not included in analyses for that particular outcome.

The adjusted RR with 95% confidence interval for each complication–outcome pair was calculated using a Poisson regression model with log link and robust error variance^26^. Non-linearity of continuous variables was addressed with restricted cubic splines (3 knots). Subsequently, the adjusted PAF was estimated separately for each of the imputed data sets for the pairs with a significant association and pooled according to Rubin’s rules^27^. The adjusted PAF represents the anticipated percentage reduction in the frequency of an adverse outcome if a certain complication could be completely prevented in the study population.

Based on previous studies and expert consensus, models were adjusted for confounders, including age, sex, BMI, ASA grade, co-morbidities (as listed in *Table S1*), previous abdominal or thoracic surgery, open, converted, or minimally invasive surgery, extent of lymphadenectomy, total or subtotal gastrectomy, multivisceral resection, T and N categories, neoadjuvant treatment, number of complications (one or multiple), and hospital region, unless otherwise stated. As the frequency of more than two complications per patient was low (*Fig. S1*), this variable was dichotomized. Ten (non-)events of the investigated outcome were required per category of a variable. In the case of an insufficient number of events for the entire model, stepwise forward selection was performed based on the Akaike information criterion. The severity of complications, in accordance with the Clavien–Dindo (CD) classification, was not included in the models, as several CD grades are incorporated in the study outcomes (for example CD grade III and CD grade V are defined as re-intervention and death respectively).

Subgroup analyses were performed for patients from East Asia and Europe/America to assess regional differences. Further sensitivity analyses were performed, while adjusting for the occurrence of multiple complications. Some complications may cause other studied complications. As anastomotic leakage can lead to pulmonary complications, the PAF for pulmonary complications was also estimated adding anastomotic leakage as a variable to the model for 30-day mortality^28^. As abdominal collection was, by definition, excluded from coexisting with anastomotic leakage (definitions listed in *Table 1*), no sensitivity analysis was conducted. The correlation of complications was also assessed with Spearman’s correlation coefficient and visualized in a correlation plot to evaluate whether other sensitivity analyses were appropriate.

**Table 1:** Postoperative complications and adverse outcomes (*n* = 7829)

Variables	Values
**Postoperative complications**	
Pulmonary complications*	436 (5.6); missing 0 (0.0)
Anastomotic leakage^†^	363 (4.6); missing 0 (0.0)
Abdominal collection^‡^	301 (3.8); missing 0 (0.0)
Ileus/motility disorder^§^	183 (2.3); missing 0 (0.0)
Fistula (pancreatic and lymphatic)	188 (2.4); missing 0 (0.0)
Surgical-site infection	123 (1.6); missing 0 (0.0)
Cardiac complications^¶^	97 (1.2); missing 0 (0.0)
Intra-abdominal bleeding	91 (1.2); missing 0 (0.0)
Stenosis of anastomosis	85 (1.1); missing 0 (0.0)
Luminal bleeding	62 (0.8); missing 0 (0.0)
Ischaemia^#^	39 (0.5); missing 0 (0.0)
Renal insufficiency**	30 (0.4); missing 0 (0.0)
Other^††^	430 (5.4); missing 0 (0.0)
Total proportion of patients with complications	1884 (24.1); missing 0 (0.0)
**Adverse outcomes**	
Escalation of care^‡‡^	332 (4.2); missing 272 (3.5)
Reoperation^§§^	377 (4.8); missing 2554 (32.6)
Readmission	437 (5.6); missing 2828 (36.1)
Mortality (30-day)	94 (1.2); missing 0 (0.0)
Prolonged hospital stay^¶¶^	2073 (26.5); missing 258 (3.3)

Values are *n* (%). *Pneumonia, pleural effusion, respiratory failure, pneumothorax, and/or pulmonary embolism. ^†^Any clinically or radiologically proven anastomotic leakage. ^‡^Intra-abdominal abscess and/or abdominal collection without anastomotic leak. ^§^Postoperative paralytic ileus, mechanical ileus, or delayed gastric emptying. ^¶^Supraventricular and ventricular arrhythmia, myocardial infarction, and/or heart failure. ^#^Postoperative bowel perforation or necrosis. **Acute renal failure, requiring dialysis. ^††^Includes vascular, urinary, and gastrointestinal complications, hepatic insufficiency, cholecystitis, and other types of infection. ^‡‡^Unplanned readmission to a unit of higher surveillance (either an intermediate care unit or an ICU). ^§§^Surgical intervention under general anaesthesia. ^¶¶^Greater than the 75th percentile, stratified for surgical access.

Categorical variables are presented as *n* (%). Continuous variables are presented as mean(s.d.), unless their distribution was skewed. The threshold for significance was set at *P* < 0.050 (two-sided). All statistical analysis was performed in R (R Core Team, R Foundation for Statistical Computing, Boston, MA, USA; version 4.3.2) using the ‘AF’ package, which allows for confounder-adjusted estimation of PAFs for cohort studies, and the ‘mice’, ‘lme4’, ‘sandwich’, and ‘mitools’ packages^29^.

## Results

In total, 9662 patients were identified in the database, of which 7829 (4042 patients from East Asia and 3787 patients from Europe/America) were included for analysis (*Fig. S2*). The mean(s.d.) age was 65.8 (12.4) years and 63.6% of patients was male. Most patients presented with a cT3 tumour (24.1%) and clinical node-negative disease (32.0%). Neoadjuvant therapy was administered in 25.4% of patients. Of all patients, 53.9% underwent minimally invasive resection. Patient and tumour characteristics are shown in *Table S1*.

The most common adverse events were pulmonary complications (5.6%), anastomotic leakage (4.6%), and abdominal collection (3.8%) (*Table 1*). The 30-day mortality rate was 1.2%. Rates of escalation of care, reoperation, and readmission were 4.2%, 4.8%, and 5.6% respectively. The rate of prolonged hospital stay was 26.5%.

### Study outcomes

See *Tables 2–6*. Anastomotic leakage, cardiac complications, and pulmonary complications had the greatest impact on 30-day mortality, with risk-adjusted PAF estimates of 26.6% (95% c.i. 14.5% to 38.6%), 18.7% (95% c.i. 9.4% to 28.0%), and 15.6% (95% c.i. 12.0% to 30.0%) respectively. Anastomotic leakage and pulmonary complications had the greatest impact on escalation of care, with PAF estimates of 26.3% (95% c.i. 20.6% to 32.0%) and 18.4% (95% c.i. 11.7% to 25.2%) respectively, whereas anastomotic leakage and intra-abdominal bleeding had the greatest impact on reoperation (PAF 31.6% (95% c.i. 26.4% to 36.9%) and 8.5% (95% c.i. 5.5% to 11.5%) respectively). All studied complications contributed to prolonged hospital stay, except for cardiac complications and renal insufficiency. The effect of complications on readmission was relatively small; anastomotic leakage (PAF 4.8% (95% c.i. 1.5% to 8.3%)), abdominal collection (PAF 3.4% (95% c.i. 1.0% to 5.7%)), ileus/motility disorder (PAF 3.8% (95% c.i. 1.5% to 6.1%)), and surgical-site infection (PAF 1.8% (95% c.i. 0.2% to 3.4%)) had an impact on readmission. Hence, approximately 15.9% of the readmissions were attributable to complications.

**Table 2:** Risk-adjusted associations and PAF between escalation of care and complications after gastrectomy (*n* = 7557)

Postoperative complication	No. with/without escalation of care	Adjusted RR (95% c.i.)	*P*	Adjusted PAF (95% c.i.), %	*P*
Pulmonary complications	117/298	2.21 (1.63,2.98)	<0.001	18.4 (11.7,25.2)	<0.001
Anastomotic leakage	121/214	4.19 (3.17,5.55)	<0.001	26.3 (20.6,32.0)	<0.001
Abdominal collection	19/274	1.62 (1.01,2.61)	0.049	3.3 (0.8,6.3)	0.013
Ileus/motility disorder	16/162	1.34 (0.80,2.26)	0.255	–	–
Fistula (pancreatic and lymphatic)	16/164	1.62 (0.96,2.73)	0.075	–	–
Surgical-site infection	12/101	0.94 (0.52,1.71)	0.779	–	–
Cardiac complications	49/44	2.14 (1.51,3.05)	<0.001	7.7 (4.2,11.1)	<0.001
Intra-abdominal bleeding	36/49	2.27 (1.56,3.30)	<0.001	5.7 (2.7,8.8)	<0.001
Stenosis of anastomosis	5/80	1.01 (0.41,2.43)	0.963	–	–
Luminal bleeding	19/40	2.19 (1.36,3.54)	0.002	3.1 (0.9,5.2)	0.005
Ischaemia	14/23	2.27 (1.30,3.96)	0.004	2.3 (0.3,4.3)	0.025
Renal insufficiency	9/19	0.93 (0.47,1.84)	0.808	–	–

RR, relative risk; PAF, population attributable fraction.

**Table 3:** Risk-adjusted associations and PAF between reoperation and complications after gastrectomy (*n* = 5275)

Postoperative complication	No. with/without reoperation	Adjusted RR (95% c.i.)	*P*	Adjusted PAF (95% c.i.), %	*P*
Pulmonary complications	75/288	0.84 (0.62,1.12)	0.263	–	–
Anastomotic leakage	149/131	5.87 (4.56,7.56)	<0.001	31.6 (26.4,36.9)	<0.001
Abdominal collection	25/153	1.09 (0.60,1.40)	0.698	–	–
Ileus/motility disorder	35/101	1.66 (1.23,2.58)	0.002	4.1 (1.3,6.9)	0.005
Fistula (pancreatic and lymphatic)	16/155	1.72 (1.02,2.90)	0.040	3.1 (0.8,5.4)	0.008
Surgical-site infection	15/70	1.56 (0.91,2.67)	0.102	–	–
Cardiac complications	25/62	1.10 (0.71,1.71)	0.285	–	–
Intra-abdominal bleeding	47/28	3.60 (2.58,5.02)	<0.001	8.5 (5.5,11.5)	<0.001
Stenosis of anastomosis	6/9	3.97 (1.73,9.07)	0.001	1.1 (−0.0,2.2)	0.064
Luminal bleeding	16/31	1.94 (1.15,3.27)	0.015	2.0 (0.2,3.8)	0.027
Ischaemia	23/10	4.26 (2.74,6.62)	<0.001	4.7 (2.6,6.8)	<0.001
Renal insufficiency	9/16	1.08 (0.57,2.06)	0.807	–	–

RR, relative risk; PAF, population attributable fraction.

**Table 4:** Risk-adjusted associations and PAF between prolonged hospital stay and complications after gastrectomy (*n* = 7571)

Postoperative complication	No. with/without prolonged hospital stay	Adjusted RR (95% c.i.)	*P*	Adjusted PAF (95% c.i.), %	*P*
Pulmonary complications	263/144	1.54 (1.32,1.81)	<0.001	4.0 (2.9,5.1)	<0.001
Anastomotic leakage	290/46	2.46 (2.12,2.84)	<0.001	7.5 (6.4,8.7)	<0.001
Abdominal collection	245/44	2.42 (2.09,2.80)	<0.001	6.7 (5.7,7.8)	<0.001
Ileus/motility disorder	131/48	2.10 (1.75,2.52)	<0.001	3.1 (2.3,3.8)	<0.001
Fistula (pancreatic and lymphatic)	123/60	1.84 (1.52,2.24)	<0.001	2.6 (1.9,3.4)	<0.001
Surgical-site infection	72/47	1.44 (1.12,1.83)	0.003	1.1 (0.5,1.7)	<0.001
Cardiac complications	45/43	1.12 (0.82,1.52)	0.513	–	–
Intra-abdominal bleeding	59/30	1.36 (1.04,1.79)	0.028	0.7 (0.2,1.2)	0.016
Stenosis of anastomosis	58/20	1.99 (1.53,2.60)	<0.001	1.5 (1.0,2.0)	<0.001
Luminal bleeding	40/20	1.65 (1.16,2.18)	0.003	0.7 (0.3,1.1)	<0.001
Ischaemia	28/10	1.43 (0.97,2.09)	0.042	0.5 (0.1,0.8)	0.012
Renal insufficiency	18/11	1.00 (0.63,1.61)	0.957	–	–

RR, relative risk; PAF, population attributable fraction.

**Table 5:** Risk-adjusted associations and PAF between 30-day mortality and complications after gastrectomy (*n* = 7829)

Postoperative complication	No. with/without mortality	Adjusted RR (95% c.i.)*	*P*	Adjusted PAF (95% c.i.), %*	*P*
Pulmonary complications	33/403	1.83 (1.06,3.18)	0.032	15.6 (12.0,30.0)	0.033
Anastomotic leakage	36/328	3.56 (2.16,5.84)	<0.001	26.6 (14.5,38.6)	<0.001
Abdominal collection	2/299	0.18 (0.94,23.36)	0.067	–	–
Ileus/motility disorder	4/179	0.71 (0.51,3.92)	0.499	–	–
Fistula (pancreatic and lymphatic)	3/185	0.39 (0.12,1.26)	0.108	–	–
Surgical-site infection	0/122	–	–	–	–
Cardiac complications	23/74	4.96 (2.83,8.70)	<0.001	18.7 (9.4,28.0)	<0.001
Intra-abdominal bleeding	7/84	1.55 (0.64,3.20)	0.374	–	–
Stenosis of anastomosis	1/84	0.42 (0.06,2.97)	0.769	–	–
Luminal bleeding	2/60	1.10 (0.22,3.70)	0.882	–	–
Ischaemia	11/28	6.10 (3.11,11.97)	<0.001	9.7 (3.3,16.1)	0.003
Renal insufficiency	7/23	2.87 (1.28,6.40)	0.014	4.9 (−0.0,9.8)	0.053

^*^Adjusted for: age, sex, renal co-morbidity, N category, neoadjuvant chemotherapy, total or subtotal gastrectomy, and number of complications (one or multiple). RR, relative risk; PAF, population attributable fraction.

**Table 6:** Risk-adjusted associations and PAF between readmission and complications after gastrectomy (*n* = 5001)

Postoperative complication	No. with/without readmission	Adjusted RR (95% c.i.)	*P*	Adjusted PAF (95% c.i.), %	*P*
Pulmonary complications	54/288	1.08 (0.76,1.51)	0.678	–	–
Anastomotic leakage	55/197	1.61 (1.16,2.22)	0.005	4.9 (1.5,8.3)	0.005
Abdominal collection	33/137	2.02 (1.39,2.91)	<0.001	3.4 (1.0,5.7)	0.005
Ileus/motility disorder	31/100	1.95 (1.33,2.86)	<0.001	3.8 (1.5,6.1)	0.001
Fistula (pancreatic and lymphatic)	23/140	1.25 (0.81,1.94)	0.314	–	–
Surgical-site infection	16/60	2.23 (1.33,3.75)	0.002	1.8 (0.3,3.4)	0.020
Cardiac complications	9/73	2.14 (1.06,4.34)	0.039	2.0 (0.3,3.6)	0.018
Intra-abdominal bleeding	13/56	1.02 (0.57,1.83)	0.850	–	–
Stenosis of anastomosis	4/11	1.91 (0.79,4.64)	0.503	–	–
Luminal bleeding	13/31	2.14 (1.21,3.79)	0.058	–	–
Ischaemia	6/24	1.25 (0.55,2.84)	0.591	–	–
Renal insufficiency	2/21	2.61 (0.56,10.52)	0.277	–	–

RR, relative risk; PAF, population attributable fraction.

### East Asia *versus* Europe/America

Patient characteristics, complications, and adverse outcomes according to region are shown in *Tables S2*, *S3*. For escalation of care, the PAF estimate of pulmonary complications in East Asian patients was 28.4% (95% c.i. 16.2% to 40.5%), which was higher than that in Western patients (PAF 14.2% (95% c.i. 6.5% to 21.9%)) (*Table S4*). Intra-abdominal bleeding had a greater impact on patients in East Asia (PAF 13.6% (95% c.i. 5.8% to 21.3%) compared with patients in the West (PAF 3.4% (95% c.i. 0.3% to 6.5%). Regarding prolonged hospital stay, no substantial differences were observed between regions, except for the PAF of anastomotic leakage and pulmonary complications, which were higher in Western patients (PAF 12.2% (95% c.i. 10.0% to 14.3%) *versus* 3.7% (95% c.i. 2.6% to 4.8%) and PAF 6.2% (95% c.i. 3.9% to 8.4%) *versus* 2.2% (95% c.i. 1.3% to 3.1%)) (*Table S5*). Data were insufficient for subgroup analyses for 30-day mortality, readmission, and reoperation due to missing adverse outcomes or a low number of events in patients in East Asia. Subgroup analyses for these outcomes in European/American patients are shown in *Tables S6–S8*.

### Sensitivity analysis

Overall, 75 of 363 patients with anastomotic leakage also suffered from pulmonary complications. Adding pulmonary complications to the models for 30-day mortality resulted in a PAF estimate of 26.6% (95% c.i. 14.6% to 38.6%), similar to the PAF without adjustment for pulmonary complications. There were no strong correlations between different complications (*Fig. S3*) and therefore no further sensitivity analyses were performed.

## Discussion

Anastomotic leakage and pulmonary complications had the highest overall impact on adverse outcomes, specifically on escalation of care, reoperation, and 30-day mortality. Prevention of anastomotic leakage would result in estimated one-third reductions in 30-day mortality, escalation of care, and reoperation, and eliminating pulmonary complications would result in approximately one-fifth reductions in 30-day mortality and escalation of care. Preventing these complications would most substantially reduce hospital costs^10^. There was less effect of the investigated complications on readmission and prolonged hospital stay.

The causes and onset of complications after gastrectomy are multifaceted. A number of factors are known to influence pulmonary complication rates and can be optimized through preoperative and postoperative interventions, which include cessation of smoking, cardiopulmonary pre-conditioning, adequate pain management, early mobilization, and breathing exercises^30^. The postoperative quality initiative (POQI) issued a consensus statement summarizing these interventions to reduce pulmonary complications after upper gastrointestinal surgery^30^. Broad implementation of these measures may reduce rates of mortality and escalation of care. For anastomotic leakage, nutritional status can be optimized to improve patient outcomes, but many of the known risk factors of anastomotic leakage are patient-related and cannot be amended before surgery^31^. Other factors such as prolonged operating time, blood loss, anastomotic technique, and surgeon proficiency are also associated with leakage, as reflected by the learning curve of gastrectomy^31,32^. Additionally, appropriate anaesthetic management aimed at prevention of hypotension, a low pH during surgery, and the use of vasopressors seem equally important^33^. Centralized or dedicated upper gastrointestinal care and adequate proctoring may enable optimization of these surgical and anaesthetic aspects^34^.

Most investigated complications had an impact on prolonged hospital stay after gastrectomy, with anastomotic leakage and abdominal collection being the largest contributors^35^. In contrast, merely four complications were associated with readmission. Contrary to expectations, less than one-fifth of the readmissions were attributable to postoperative complications. Previous studies have reported that approximately 80% of the complications after gastrointestinal surgery are diagnosed during initial admission, with only half of patients who experience complications after discharge being readmitted^22^. Notably, nosocomial infections (such as urinary, clostridium, or other gastrointestinal infections), which often lead to readmission, were not included in the present analysis^36^. Additionally, data on specific reasons for readmission were lacking. Therefore, causes such as poor intake or dehydration, previously reported to be common reasons for readmission, could not be assessed^37,38^.

The PAF has been used previously to identify complications with the greatest impact on several outcomes (such as mortality, readmission, and prolonged hospital stay) in patients with gastric cancer in the Netherlands^18^. In the present study, it was found that cardiac complications had a greater impact on 30-day mortality compared with Gertsen *et al*.^18^. This discrepancy may be attributed to differences in the definition for cardiac complications used in the Western cohort of the present study, which only included major cardiac complications. Cardiac complications resulted in a five-fold increased risk of 30-day mortality in the present study, compared with a three-fold increased risk of 30-day mortality in Gertsen *et al*.^18^. Notably, pancreatic fistulas were not included in the PAF analysis in Gertsen *et al*.^18^. While fistulas can be challenging to manage in the clinic, they had a low overall impact on the adverse outcomes. Consistent with the present findings, Gertsen *et al*.^18^ also identified anastomotic leakage and pulmonary complications to have the greatest impact on several adverse outcomes. However, Gertsen *et al*.^18^ only included patients from the Dutch Upper Gastrointestinal Cancer Audit (DUCA), although the majority of gastric cancer patients originate from the East, as the incidence of gastric adenocarcinoma is far higher in countries such as Japan, Korea, and China, where lower complication rates are typically achieved^39^.

Subgroup analysis per region showed variation in the impact of complications. Although the rate of pulmonary complications in East Asian patients was lower, pulmonary complications still had a major impact on escalation of care, having twice the impact compared with Western patients. Similarly, intra-abdominal bleeding and abdominal collection had higher PAFs in East Asian patients. These differences underline that not only patient and treatment characteristics (which were adjusted for) but also healthcare system factors influence the onset of clinical adverse outcomes. Several studies have reported that organizational aspects such as low nurse staffing levels, poor access to and use of radiology, and unstructured emergency operation schedules contribute to mortality and reoperation^40–42^. A centre’s resources and infrastructure influence morbidity management and its ability to manage and ‘rescue’ complications. Therefore, region- or country-specific strategies are likely warranted to effectively reduce complications and mitigate their associated burden and downstream effects. To better understand the occurrence of these adverse outcomes, future studies should incorporate system- and organizational-level factors that contribute to their development.

This study has several limitations. Inherent to using the PAF, it was assumed that the effects of the complications were at least in part responsible for the adverse outcomes. As data on the day of occurrence of outcomes were lacking, the authors were unable to verify the sequence of events. For patients who were reoperated on, the possibility exists that a complication was caused by the second intervention. For two of the five study outcomes more than a quarter of the values were missing, limiting the number of patients analysed for these adverse outcomes and preventing subgroup analysis per region for three of five investigated outcomes. The number of patients included per centre varied, with a higher caseload for East Asian centres^5^. The authors adjusted for hospital region and other potential differences, as the Western population typically has more advanced disease, more co-morbidities, a higher BMI, and is older. However, the possibility of unmeasured confounding remains. Finally, two different forms were used to register complications. This meant that it was necessary to group several complications. This was particularly relevant for pulmonary complications, as it was not possible to categorize these into more specific complications.

## Collaborators

**The GastroBenchmark Consortium**

Hidde Overtoom (Department of Surgery, Erasmus University Medical Centre, Rotterdam, The Netherlands); Ines Gockel (Department of Visceral, Transplant, Thoracic and Vascular Surgery, University Hospital of Leipzig, Leipzig, Germany); René Thieme (Department of Visceral, Transplant, Thoracic and Vascular Surgery, University Hospital of Leipzig, Leipzig, Germany); Ewen A. Griffiths (Department of Upper GI Surgery, Queen Elizabeth Hospital, University Hospitals Birmingham NHS Foundation Trust, Birmingham, UK); William Butterworth (Department of Upper GI Surgery, Queen Elizabeth Hospital, University Hospitals Birmingham NHS Foundation Trust, Birmingham, UK); Henrik Nienhüser (Klinik für Allgemein-, Viszeral- und Transplantationschirurgie, Universitätsklinikum Heidelberg, Heidelberg, Germany); Beat Müller (Klinik für Allgemein-, Viszeral- und Transplantationschirurgie, Universitätsklinikum Heidelberg, Heidelberg, Germany); Nerma Crnovrsanin (Klinik für Allgemein-, Viszeral- und Transplantationschirurgie, Universitätsklinikum Heidelberg, Heidelberg, Germany); Felix Nickel (Department of General, Visceral, and Thoracic Surgery, University Medical Centre Hamburg-Eppendorf, Hamburg, Germany); Suzanne S. Gisbertz (Department of Surgery, Amsterdam UMC, University of Amsterdam, & Cancer Centre Amsterdam, Cancer Treatment and Quality of Life, Amsterdam, The Netherlands); Mark I. van Berge Henegouwen (Department of Surgery, Amsterdam UMC, University of Amsterdam, & Cancer Centre Amsterdam, Cancer Treatment and Quality of Life, Amsterdam, The Netherlands); Philip H. Pucher (Department of Surgery, Queen Alexandra Hospital, Portsmouth Hospitals NHS Trust, Portsmouth, UK); Kashuf Khan (Department of Surgery, Queen Alexandra Hospital, Portsmouth Hospitals NHS Trust, Portsmouth, UK); Asif Chaudry (The Royal Marsden NHS Foundation Trust, Chelsea, London, SW3 6JJ, UK); Pranav H. Patel (The Royal Marsden NHS Foundation Trust, Chelsea, London, SW3 6JJ, UK); Manuel Pera (Section of Gastrointestinal Surgery, Hospital Universitario del Mar, Universitat Autònoma de Barcelona, Barcelona, Spain); Mariagiulia Dal Cero (Section of Gastrointestinal Surgery, Hospital Universitario del Mar, Universitat Autònoma de Barcelona, Barcelona, Spain); Carlos Garcia (Hospital San Borja Arriarán, Av. Sta. Rosa 1234, Santiago, Región Metropolitana, Chile); Guillermo Martinez Salinas (Hospital San Borja Arriarán, Av. Sta. Rosa 1234, Santiago, Región Metropolitana, Chile); Paulo Kassab (Gastro-oesophageal and Bariatric Surgical Division, Department of Surgery, Santa Casa of São Paulo Medical School and Hospital, São Paulo, Brazil); Osvaldo Antônio Prado Castro (Gastro-oesophageal and Bariatric Surgical Division, Department of Surgery, Santa Casa of São Paulo Medical School and Hospital, São Paulo, Brazil); Enrique Norero (Oesophagogastric Surgery Unit, Digestive Surgery Department, Hospital Dr Sotero del Rio, Pontificia Universidad Catolica de Chile, Santiago, Chile); Paul Wisniowski (Division of Upper GI and General Surgery, Keck School of Medicine, University of Southern California, 1510 San Pablo St., Health Sciences Campus, Los Angeles, USA); Luke Randall Putnam (Division of Upper GI and General Surgery, Keck School of Medicine, University of Southern California, 1510 San Pablo St., Health Sciences Campus, Los Angeles, USA); Pietro Maria Lombardi (Division of Minimally Invasive Surgical Oncology, Niguarda Cancer Centre, ASST Grande Ospedale Metropolitano Niguarda, Piazza Ospedale Maggiore, 3, 20162, Milan, Italy); Giovanni Ferrari (Division of Minimally Invasive Surgical Oncology, Niguarda Cancer Centre, ASST Grande Ospedale Metropolitano Niguarda, Piazza Ospedale Maggiore, 3, 20162, Milan, Italy); Rita Gudaityte (Department of Surgery, Hospital of Lithuanian University of Health Sciences, Eiveniu 2, Kaunas 50161, Lithuania); Almantas Maleckas (Department of Surgery, Hospital of Lithuanian University of Health Sciences, Eiveniu 2, Kaunas 50161, Lithuania); Leanne Prodehl (Department of Surgery, Charlotte Maxeke Johannesburg Academic Hospital, University of the Witwatersrand, Johannesburg, South Africa); Antonio Castaldi (Service de Chirurgie Digestive et Cancérologie Digestive, Hôpital Universitaire Carémeau, Nîmes, France); Michel Prudhomme (Service de Chirurgie Digestive et Cancérologie Digestive, Hôpital Universitaire Carémeau, Nîmes, France); Simone Giacopuzzi (Department of Surgery, University Hospital of Verona, Verona, Italy); Riccardo Rosati (Department of Surgery, San Raffaele Hospital, Milano, Italy); Francesco Puccetti (Department of Surgery, San Raffaele Hospital, Milano, Italy); Domenico D’Ugo (FONDAZIONE POLICLINICO UNIVERSITARIO GEMELLI-IRCCS, Roma, Italy); Daniel Gero (Department of Surgery & Transplantation, University Hospital Zürich, Raemistrasse 100, 8091 Zurich, Switzerland); Hyuk-Joon Lee (Department of Surgery, Seoul National University Cancer Hospital, 101 Daehak-ro Jongno-gu, Seoul, South Korea).

**The GASTRODATA Consortium**

Guillaume Piessen (Department of Surgery, University Hospital of Lille, Lille, France); Justine Lerooy (Department of Surgery, University Hospital of Lille, Lille, France); Johanna Wilhelmina van Sandick (Department of Surgical Oncology, The Netherlands Cancer Institute—Antoni van Leeuwenhoek Hospital, Postbus, 90203 1006 BE, Amsterdam, The Netherlands); Suzanne S. Gisbertz (Department of Surgery, Amsterdam UMC, University of Amsterdam, & Cancer Centre Amsterdam, Cancer Treatment and Quality of Life, Amsterdam, The Netherlands); Mark I. van Berge Henegouwen (Department of Surgery, Amsterdam UMC, University of Amsterdam, & Cancer Centre Amsterdam, Cancer Treatment and Quality of Life, Amsterdam, The Netherlands); Jessie Elliott (Department of Surgery, St. James’s Hospital, Trinity College Dublin, Dublin, Ireland); Paolo Morgagni (GB Morgagni-L Pierantoni Hospital, Forlì, Italy); Arnulf H. Hölscher (Contilia Centre for Oesophageal Diseases, Elisabeth Hospital Essen, West German Tumour Centre, University Medicine Essen, Germany); Martin Hemmerich (Contilia Centre for Oesophageal Diseases, Elisabeth Hospital Essen, West German Tumour Centre, University Medicine Essen, Germany); Stefan Mönig (Department of Surgery, University Hospital of Geneva, Geneva, Switzerland); Mickael Chevallay (Department of Surgery, University Hospital of Geneva, Geneva, Switzerland); Piotr Kołodziejczyk (Department of Surgery, Jagiellonian University, Kraków, Poland); Henk Hartgrink (Leiden University Medical Centre, Leiden, The Netherlands); Paulo Matos da Costa (Faculdade de Medicina, Universidade de Lisboa; Lisboa, Portugal); Filipe Castro Borges (Faculdade de Medicina, Universidade de Lisboa; Lisboa, Portugal); Andrew Davies (Department of Surgery, Guy’s & St Thomas’ NHS Foundation Trust, London, UK); Cara Baker (Department of Surgery, Guy’s & St Thomas’ NHS Foundation Trust, London, UK); William Allum (The Royal Marsden NHS Foundation Trust, Chelsea, London, SW3 6JJ, UK); Sacheen Kumar (The Royal Marsden NHS Foundation Trust, Chelsea, London, SW3 6JJ, UK); Wojciech Polkowski (Medical University of Lublin, Lublin, Poland); Karol Rawicz-Pruszyński (Medical University of Lublin, Lublin, Poland); Uberto Fumagalli Romario (Digestive Surgery, European Institute of Oncology, IRCCS, Milano, Italy); Stefano De Pascale (Digestive Surgery, European Institute of Oncology, IRCCS, Milano, Italy); Antonio Tarasconi (Department of Surgery, University Hospital of Brescia, Brescia, Italy); Daniel Reim (Department of Surgery, TUM School of Medicine, Technical University of Munich, Germany); Ilaria Pergolini (Department of Surgery, TUM School of Medicine, Technical University of Munich, Germany); Lucio Lara Santos (Department of Surgery, Portuguese Institute of Oncology, Porto, Portugal); Pedro Carvalho Martins (Department of Surgery, Portuguese Institute of Oncology, Porto, Portugal); Alberto Biondi (FONDAZIONE POLICLINICO UNIVERSITARIO GEMELLI-IRCCS, Roma, Italy); Riccardo Rosati (Department of Surgery, San Raffaele Hospital, Milano, Italy); Maurizio Degiuli (Department of Surgical Oncology and Digestive Surgery, San Luigi University Hospital Orbassano, School of Medicine, University of Torino, Torino, Italy); Rossella Reddavid (Department of Surgical Oncology and Digestive Surgery, San Luigi University Hospital Orbassano, School of Medicine, University of Torino, Torino, Italy); Wojciech Kielan (Wroclaw Medical University, Wroclaw, Poland); Paul Magnus Schneider (Digestive Oncology Tumour Centre and Oesophageal Cancer Centre, Hirslanden Medical Centre, Zurich, Switzerland); Thomas Murphy (Mercy University Hospital, Cork, Ireland).

## Supplementary Material

znaf043_Supplementary_Data

## Data Availability

The data sets used and analysed will be available upon reasonable request.
