# Supplementary material for: Impact of postoperative complications on clinical outcomes after gastrectomy for cancer: multicentre study
Source: Br J Surg. 2025 Mar 28;112(4):znaf043. doi: 10.1093/bjs/znaf043 (PMC11953074; doi:10.1093/bjs/znaf043)

**Impact of postoperative complications on clinical outcomes after gastrectomy for cancer: multicentre study**

Sander JM van Hootegem^1^, Margrietha van der Linde^2^, Marcel A Schneider^3^, Jeesun Kim^4^, Felix Berlth^5,6^, Yutaka Sugita^7^, Peter P Grimminger^5^, Gian Luca Baiocchi^8^, Giovanni De Manzoni^9^, Maria Bencivenga^9^, Suzanne Gisbertz^10^, Souya Nunobe^7^, Han-Kwang Yang^4^, Christian A Gutschow^3^, Hester F Lingsma^2^, Bas PL Wijnhoven^1^, The GastroBenchmark & GASTRODATA consortiums†

^1^ Department of Surgery, Erasmus Medical Center, Netherlands

^2^ Department of Public Health, Erasmus Medical Center, Netherlands

^3^ Department of Surgery & Transplantation, University Hospital Zürich, Switzerland

^4^ Department of Surgery, Seoul National University Cancer Hospital, Korea

^5^ Department of General-, Visceral- and Transplant Surgery, University Medical Center Mainz, Germany

^6^ Department of Surgery, University Hospital of Tuebingen, Germany

^7^ Department of Gastroenterological Surgery, Cancer Institute Hospital of the Japanese Foundation for Cancer Research, Japan

^8^ Department of Surgery, University Hospital of Brescia, Italy

^9^ Department of Surgery, University Hospital of Verona, Italy

^10^ Department of Surgery, Amsterdam UMC, University of Amsterdam, Amsterdam, The Netherlands

^11^ Cancer Centre Amsterdam, Cancer Treatment and Quality of Life, Amsterdam, The Netherlands

† Listed under the header Collaborators in supplementary appendix.

**Corresponding author:**

SJM van Hootegem

Department of Surgery, Erasmus MC, University Medical Center

P.O. Box 2040, 3000CA Rotterdam, The Netherlands

ORCID ID: <https://orcid.org/0000-0002-0220-028X>

**Supplementary Materials - Index**

**Supplementary appendixes.**

Collaborators page 3-6

Participating centres page 7-8

**Supplementary Figures and Tables.**

Supplementary tables page 9-20

Supplementary figures page 21-24

**Supplement 1**

**Collaborators**

*GastroBenchmark Consortium*

Hidde Overtoom (Department of Surgery, Erasmus University Medical Center, Rotterdam, The Netherlands); Ines Gockel (Department of Visceral, Transplant, Thoracic and Vascular Surgery, University Hospital of Leipzig, Leipzig, Germany); René Thieme (Department of Visceral, Transplant, Thoracic and Vascular Surgery, University Hospital of Leipzig, Leipzig, Germany); Ewen A. Griffiths (Department of Upper GI Surgery, Queen Elizabeth Hospital, University Hospitals Birmingham NHS Foundation Trust, Birmingham, UK); William Butterworth (Department of Upper GI Surgery, Queen Elizabeth Hospital, University Hospitals Birmingham NHS Foundation Trust, Birmingham, UK); Henrik Nienhüser (Klinik für Allgemein-, Viszeral- und Transplantationschirurgie, Universitätsklinikum Heidelberg, Heidelberg, Germany); Beat Müller (Klinik für Allgemein-, Viszeral- und Transplantationschirurgie, Universitätsklinikum Heidelberg, Heidelberg, Germany); Nerma Crnovrsanin (Klinik für Allgemein-, Viszeral- und Transplantationschirurgie, Universitätsklinikum Heidelberg, Heidelberg, Germany); Felix Nickel (Department of General, Visceral, and Thoracic Surgery, University Medical Center Hamburg-Eppendorf, Hamburg, Germany); Suzanne S. Gisbertz (Department of Surgery, Amsterdam UMC, University of Amsterdam, & Cancer Center Amsterdam, Cancer Treatment and Quality of Life, Amsterdam, The Netherlands ); Mark I. van Berge Henegouwen (Department of Surgery, Amsterdam UMC, University of Amsterdam, & Cancer Center Amsterdam, Cancer Treatment and Quality of Life, Amsterdam, The Netherlands); Philip H. Pucher (Department of Surgery, Queen Alexandra Hospital, Portsmouth Hospitals NHS Trust, Portsmouth, UK); Kashuf Khan (Department of Surgery, Queen Alexandra Hospital, Portsmouth Hospitals NHS Trust, Portsmouth, UK); Asif Chaudry (The Royal Marsden NHS Foundation Trust, Chelsea, London, SW3 6JJ, UK); Pranav H. Patel (The Royal Marsden NHS Foundation Trust, Chelsea, London, SW3 6JJ, UK); Manuel Pera (Section of Gastrointestinal Surgery, Hospital Universitario del Mar, Universitat Autònoma de Barcelona, Barcelona, Spain); Mariagiulia Dal Cero (Section of Gastrointestinal Surgery, Hospital Universitario del Mar, Universitat Autònoma de Barcelona, Barcelona, Spain); Carlos Garcia (Hospital San Borja Arriarán, Av. Sta. Rosa 1234, Santiago, Región Metropolitana, Chile); Guillermo Martinez Salinas (Hospital San Borja Arriarán, Av. Sta. Rosa 1234, Santiago, Región Metropolitana, Chile); Paulo Kassab (Gastroesophageal and Bariatric Surgical Division, Department of Surgery, Santa Casa of São Paulo Medical School and Hospital, São Paulo, Brazil); Osvaldo Antônio Prado Castro (Gastroesophageal and Bariatric Surgical Division, Department of Surgery, Santa Casa of São Paulo Medical School and Hospital, São Paulo, Brazil); Enrique Norero (Esophagogastric Surgery Unit, Digestive Surgery Department, Hospital Dr Sotero del Rio, Pontificia Universidad Catolica de Chile, Santiago, Chile); Paul Wisniowski (Division of Upper GI and General Surgery, Keck School of Medicine, University of Southern California, 1510 San Pablo St., Health Sciences Campus, Los Angeles, USA); Luke Randall Putnam (Division of Upper GI and General Surgery, Keck School of Medicine, University of Southern California, 1510 San Pablo St., Health Sciences Campus, Los Angeles, USA); Pietro Maria Lombardi (Division of Minimally Invasive Surgical Oncology, Niguarda Cancer Center, ASST Grande Ospedale Metropolitano Niguarda, Piazza Ospedale Maggiore, 3, 20162, Milan, Italy); Giovanni Ferrari (Division of Minimally Invasive Surgical Oncology, Niguarda Cancer Center, ASST Grande Ospedale Metropolitano Niguarda, Piazza Ospedale Maggiore, 3, 20162, Milan, Italy); Rita Gudaityte (Department of Surgery, Hospital of Lithuanian University of Health Sciences, Eiveniu 2, Kaunas 50161, Lithuania); Almantas Maleckas (Department of Surgery, Hospital of Lithuanian University of Health Sciences, Eiveniu 2, Kaunas 50161, Lithuania); Leanne Prodehl (Department of Surgery, Charlotte Maxeke Johannesburg Academic Hospital, University of the Witwatersrand, Johannesburg, South Africa); Antonio Castaldi (Service de Chirurgie Digestive et Cancérologie Digestive, Hôpital Universitaire Carémeau, Nîmes, France); Michel Prudhomme (Service de Chirurgie Digestive et Cancérologie Digestive, Hôpital Universitaire Carémeau, Nîmes, France); Simone Giacopuzzi (Department of Surgery, University Hospital of Verona, Verona, Italy); Riccardo Rosati (Department of Surgery, San Raffaele Hospital, Milano, Italy); Francesco Puccetti (Department of Surgery, San Raffaele Hospital, Milano, Italy); Domenico D'Ugo (FONDAZIONE POLICLINICO UNIVERSITARIO GEMELLI-IRCCS, Roma, Italy); Daniel Gero (Department of Surgery & Transplantation, University Hospital Zürich, Raemistrasse 100, 8091 Zurich, Switzerland); Hyuk-Joon Lee (Department of Surgery, Seoul National University Cancer Hospital, 101 Daehak-ro Jongno-gu, Seoul, South Korea).

*GASTRODATA Consortium*

Guillaume Piessen (Department of Surgery, University Hospital of Lille, Lille, France); Justine Lerooy (Department of Surgery, University Hospital of Lille, Lille, France); Johanna Wilhelmina van Sandick (Department of Surgical Oncology, The Netherlands Cancer Institute—Antoni van Leeuwenhoek Hospital, Postbus, 90203 1006 BE, Amsterdam, The Netherlands); Suzanne S. Gisbertz (Department of Surgery, Amsterdam UMC, University of Amsterdam, & Cancer Center Amsterdam, Cancer Treatment and Quality of Life, Amsterdam, The Netherlands ); Mark I. van Berge Henegouwen (Department of Surgery, Amsterdam UMC, University of Amsterdam, & Cancer Center Amsterdam, Cancer Treatment and Quality of Life, Amsterdam, The Netherlands); Jessie Elliott (Department of Surgery, St. James’s Hospital, Trinity College Dublin, Dublin, Ireland); Paolo Morgagni (GB Morgagni-L Pierantoni Hospital, Forlì, Italy); Arnulf H. Hölscher (Contilia Center for Esophageal Diseases, Elisabeth Hospital Essen, West German Tumor Center, University Medicine Essen, Germany); Martin Hemmerich (Contilia Center for Esophageal Diseases, Elisabeth Hospital Essen, West German Tumor Center, University Medicine Essen, Germany); Stefan Mönig (Department of Surgery, University Hospital of Geneva, Geneva, Switzerland); Mickael Chevallay (Department of Surgery, University Hospital of Geneva, Geneva, Switzerland); Piotr Kołodziejczyk (Department of Surgery, Jagiellonian University, Kraków, Poland); Henk Hartgrink (Leiden University Medical Center, Leiden, The Netherlands); Paulo Matos da Costa (Faculdade de Medicina, Universidade de Lisboa; Lisboa, Portugal); Filipe Castro Borges (Faculdade de Medicina, Universidade de Lisboa; Lisboa, Portugal); Andrew Davies (Department of Surgery, Guy’s & St Thomas’ NHS Foundation Trust, London, UK); Cara Baker (Department of Surgery, Guy’s & St Thomas’ NHS Foundation Trust, London, UK); William Allum (The Royal Marsden NHS Foundation Trust, Chelsea, London, SW3 6JJ, UK); Sacheen Kumar (The Royal Marsden NHS Foundation Trust, Chelsea, London, SW3 6JJ, UK); Wojciech Polkowski (Medical University of Lublin, Lublin, Poland); Karol Rawicz-Pruszyński (Medical University of Lublin, Lublin, Poland); Uberto Fumagalli Romario (Digestive Surgery, European Institute of Oncology, IRCCS, Milano, Italy); Stefano De Pascale (Digestive Surgery, European Institute of Oncology, IRCCS, Milano, Italy); Antonio Tarasconi (Department of Surgery, University Hospital of Brescia, Brescia, Italy); Daniel Reim (Department of Surgery, TUM School of Medicine, Technical University of Munich, Germany); Ilaria Pergolini (Department of Surgery, TUM School of Medicine, Technical University of Munich, Germany); Lucio Lara Santos (Department of Surgery, Portuguese Institute of Oncology, Porto, Portugal); Pedro Carvalho Martins (Department of Surgery, Portuguese Institute of Oncology, Porto, Portugal); Alberto Biondi (FONDAZIONE POLICLINICO UNIVERSITARIO GEMELLI-IRCCS, Roma, Italy); Riccardo Rosati (Department of Surgery, San Raffaele Hospital, Milano, Italy); Maurizio Degiuli (Department of Surgical Oncology and Digestive Surgery, San Luigi University Hospital Orbassano, School of Medicine, University of Torino, Torino, Italy); Rossella Reddavid (Department of Surgical Oncology and Digestive Surgery, San Luigi University Hospital Orbassano, School of Medicine, University of Torino, Torino, Italy); Wojciech Kielan (Wroclaw Medical University, Wroclaw, Poland); Paul Magnus Schneider (Digestive Oncology Tumor Center and Esophageal Cancer Center, Hirslanden Medical Center, Zurich, Switzerland); Thomas Murphy (Mercy University Hospital, Cork, Ireland).

**Participating centres**

Asia:

- Republic of Korea, Seoul, National University Cancer Hospital
- Japan, Tokyo, Cancer Institute Hospital of the JFCR

Europe:

- France, Nimes, Hôpital Universitaire Carémeau
- Germany, Heidelberg, University Hospital
- Germany, Leipzig, University Hospital
- Germany, Mainz, University Hospital
- Italy, Milano, Niguarda Hospital
- Lithuania, Kaunas, Hospital of Lithuanian University of Health Sciences
- Netherlands, Rotterdam, Erasmus Medical Center
- Spain, Barcelona, Hospital del Mar
- Switzerland, Zurich, University Hospital
- United Kingdom, Birmingham, Queen Elizabeth University Hospital
- United Kingdom, Portsmouth, Queen Alexandra Hospital
- United Kingdom, London, The Royal Marsdon (Prof. A. Chaudry)

South America:

- Brazil, São Paulo, Santa Casa
- Chile, Santiago, Hospital San Borja Arriarán
- Chile, Santiago, Hospital Dr Sotero del Rio, Pontificia Universidad Catolica de Chile

North America:

- USA, Los Angeles/CA, Keck Medical School

Africa:

- South Africa, Johannesburg, Charlotte Maxeke Johannesburg Academic Hospital/ University of the Witwatersrand

GASTRODATA Collaborative (Europe):

- France, Lille, Centre hospitaire universitaire
- Germany, Frankfurt, Agaplesion Markus Hospital
- Germany, Munich, Technical University
- Ireland, Cork, Mercy University Hospital
- Ireland, Dublin, St. James’s Hospital, Trinity College
- Italy, Brescia, University Hospital
- Italy, Forlì, GB Morgagni-L Pierantoni Hospital
- Italy, Milano, Istituto Europeo di Oncologia
- Italy, Milano, San Raffaele Hospital
- Italy, Roma, Cattolica University
- Italy, Torino, University of Torino
- Italy, Verona, University Hospital
- Netherlands, Amsterdam, Amsterdam UMC
- Netherlands, Amsterdam, The Netherlands Cancer Institute
- Netherlands, Leiden, University Medical Center
- Poland, Lublin, Medical University
- Poland, Kraków, Jagiellonian University
- Poland, Wroclaw, Medical University
- Portugal, Lisbon, University of Lisbon
- Portugal, Porto, Portuguese Institute of Oncology
- Switzerland, Geneva, University Hospital
- Switzerland, Zurich, Hirslanden Medical Center
- United Kingdom, London, St. Thomas’ NHS
- United Kingdom, London, The Royal Marsden (Prof. W. Allum)

**Supplement 2**

| **Supplementary Table 1** Patient- and treatment related characteristics (*n* = 7829) | |  |
| --- | --- | --- |
| *Variables* | **No. (%)** | **Missing (%)** |
|  |  |  |
| **Age, years** |  | 9 (0.1%) |
| Mean (SD) | 65.8 (12.4) |  |
| **Sex ratio** |  | 4 (0.1%) |
| Percentage male | 4978 (63.6%) |  |
| **BMI, kg/m2** |  | 1402 (17.7%) |
| Mean (SD) | 24.7 (4.52) |  |
| **ASA score** |  | 469 (6.0%) |
| 1 | 2148 (27.4%) |  |
| 2 | 3683 (45.8%) |  |
| 3 | 1543 (19.7%) |  |
| 4 | 86 (1.1%) |  |
| **Relevant comorbidities** |  | 0 |
| Cardiovascular | 624 (7.9%) |  |
| Pulmonary, including asthma and COPD | 135 (1.7%) |  |
| Endocrine disorder, including diabetes | 596 (7.6%) |  |
| Gastro-intestinal, including liver cirrhosis and IBD | 197 (2.5%) |  |
| Renal insuffiency (eGFR <30 or hemodialysis) | 37 (0.5%) |  |
| Immunological disorder | 38 (0.5%) |  |
| Oncological, other types of cancer | 373 (4.9%) |  |
| Multiple or other | 1360 (17.4%) |  |
| **Previous thoracic or abdominal surgery** |  | 1651 (21.1%) |
| No | 4743 (60.6%) |  |
| Minor | 1037 (13.1%) |  |
| Medium | 349 (4.5%) |  |
| Major | 63 (0.8%) |  |
| **Tumour location** |  | 233 (3.0%) |
| Antrum and Pylorus | 3044 (38.9%) |  |
| Cardia and EGJ | 697 (8.9%) |  |
| Corpus | 3089 (39.5%) |  |
| Fundus | 337 (4.3%) |  |
| Whole stomach | 293 (3.7%) |  |
| Other | 136 (1.7%) |  |
| **cT stage** |  | 2650 (33.8%) |
| cT1 | 1099 (14.0%) |  |
| cT2 | 879 (11.2%) |  |
| cT3 | 1886 (24.1%) |  |
| cT4 | 1089 (14.3%) |  |
| cTx | 226 (2.9%) |  |
| **cN stage** |  | 2646 (33.8%) |
| cN0 | 2509 (32.0%) |  |
| cN+ | 1148 (14.7%) |  |
| cN1 | 763 (9.7%) |  |
| cN2 | 220 (2.8%) |  |
| cN3 | 146 (1.9%) |  |
| cNx | 397 (5.1%) |  |
| **Neoadjuvant therapy** |  | 737 (9.4%) |
| Yes | 1990 (25.4%) |  |
| No | 5102 (65.2%) |  |
| **Type of resection** |  | 0 |
| Total gastrectomy | 2932 (37.5%) |  |
| Subtotal gastrectomy | 4897 (62.5%) |  |
| **Surgical access** |  | 0 |
| Open | 3409 (43.5%) |  |
| Laparoscopic or robot-assisted | 4220 (53.9%) |  |
| Converted | 200 (2.6%) |  |
| **Lymph node dissection** |  | 0 |
| D1 | 268 (3.4%) |  |
| D1+ | 2313 (29.5%) |  |
| D2 | 4773 (61.0%) |  |
| D2+ | 429 (5.5%) |  |
| D3 | 46 (0.6%) |  |
| **Multivisceral resection**† | 1453 (18.6%) | 0 |
| **pT category** |  | 217 (2.8%) |
| pT0 | 205 (2.6%) |  |
| pTis | 43 (0.5%) |  |
| pT1 | 2921 (37.3%) |  |
| pT2 | 996 (12.7%) |  |
| pT3 | 1874 (23.9%) |  |
| pT4 | 1555 (19.9%) |  |
| pTx | 21 (0.3%) |  |
| **pN category** |  | 425 (5.4%) |
| pN0 | 4144 (52.9%) |  |
| pN1 | 1051 (13.4%) |  |
| pN2 | 945 (12.1%) |  |
| pN3 | 1250 (16.0%) |  |
| pNx | 14 (0.2%) |  |
| *SD* Standard deviation *BMI* Body Mass Index *ASA* American Society of Anesthesiologists | | |
| † Additional resection of adjacent organ(s), including cholecystectomy. | | |

| **Supplementary Table 2** Patient- and treatment related characteristics, stratified per region. | | | |  |  |
| --- | --- | --- | --- | --- | --- |
| *Variables* | *East Asia (n = 4042)* | |  | *Europe/America (n = 3787) 37873787)* | |
|  | **No. (%)** | **Missing (%)** |  | **No. (%)** | **Missing (%)** |
| **Age, years** |  | 0 (0%) |  |  | 9 (0.2%) |
| Mean (SD) | 63.6 (12.1) |  |  | 66.5 (12.6) |  |
| **Sex** |  | 0 (0%) |  |  | 4 (0.1%) |
| Male | 2656 (65.7%) |  |  | 2332 (61.3%) |  |
| Female | 1386 (34.3%) |  |  | 1461 (38.6%) |  |
| **BMI, kg/m2** |  | 1189 (29.4%) |  |  | 213 (5.6%) |
| Mean (SD) | 23.6 (3.57) |  |  | 25.5 (4.99) |  |
| **ASA score** |  | 387 (9.6%) |  |  | 82 (2.2%) |
| 1 | 1687 (41.7%) |  |  | 461 (12.2%) |  |
| 2 | 1744 (43.1%) |  |  | 1839 (48.6%) |  |
| 3 | 213 (5.3%) |  |  | 1330 (35.1%) |  |
| 4 | 11 (0.3%) |  |  | 75 (2.0%) |  |
| **Relevant comorbidities** |  | 0 (0%) |  |  | 0 (0%) |
| Cardiovascular | 248 (6.1%) |  |  | 376 (9.9%) |  |
| Pulmonary, including asthma and COPD | 117 (2.9%) |  |  | 18 (0.5%) |  |
| Endocrine disorder, including diabetes | 340 (8.4%) |  |  | 256 (6.8%) |  |
| Gastro-intestinal, including liver cirrosis and IBD | 73 (1.8%) |  |  | 124 (3.3%) |  |
| Renal insuffiency (eGFR <30 or hemodialysis) | 11 (0.3%) |  |  | 26 (0.7%) |  |
| Immunological disorder | 28 (0.7%) |  |  | 10 (0.3%) |  |
| Oncological, other types of cancer | 143 (3.5%) |  |  | 230 (6.1%) |  |
| Multiple or other | 528 (13.1%) |  |  | 832 (22.0%) |  |
| **Previous thoracic or abdominal surgery** |  | 1282 (31.7%) |  |  | 0 (0%) |
| No | 2249 (55.6%) |  |  | 2494 (65.9%) |  |
| Minor | 420 (10.4%) |  |  | 603 (15.9%) |  |
| Medium | 84 (2.1%) |  |  | 265 (7.0%) |  |
| Major | 7 (0.2%) |  |  | 56 (1.5%) |  |
| **Tumour location** |  | 186 (4.6%) |  |  | 47 (1.2%) |
| Antrum and Pylorus | 1483 (36.7%) |  |  | 1561 (41.2%) |  |
| Cardia and EGJ | 189 (4.7%) |  |  | 508 (13.4%) |  |
| Corpus | 1553 (38.4%) |  |  | 1536 (40.6%) |  |
| Fundus | 260 (6.4%) |  |  | 77 (2.0%) |  |
| Whole stomach | 251 (6.2%) |  |  | 42 (1.1%) |  |
| Other | 120 (3.0%) |  |  | 16 (0.4%) |  |
| **cT stage** |  | 2554 (63.2%) |  |  | 96 (2.5%) |
| cT1 | 752 (18.6%) |  |  | 347 (9.2%) |  |
| cT2 | 184 (4.6%) |  |  | 695 (18.4%) |  |
| cT3 | 185 (4.6%) |  |  | 1701 (44.9%) |  |
| cT4 | 354 (8.8%) |  |  | 735 (19.4%) |  |
| cTx | 13 (0.3%) |  |  | 213 (5.6%) |  |
| **cN stage** |  | 2554 (63.2%) |  |  | 92 (2.4%) |
| cN0 | 1078 (26.7%) |  |  | 1148 (30.3%) |  |
| cN+ | 0 (0%) |  |  | 1431 (37.8%) |  |
| cN1 | 247 (6.1%) |  |  | 516 (13.6%) |  |
| cN2 | 88 (2.2%) |  |  | 132 (3.5%) |  |
| cN3 | 61 (1.5%) |  |  | 85 (2.2%) |  |
| cNx | 14 (0.3%) |  |  | 383 (10.1%) |  |
| **Neoadjuvant therapy** |  | 734 (18.2%) |  |  | 3 (0.1%) |
| Yes | 227 (5.6%) |  |  | 1763 (46.6%) |  |
| No | 3081 (76.2%) |  |  | 2021 (53.4%) |  |
| **Type of resection** |  | 0 (0%) |  |  | 0 (0%) |
| Total Gastrectomy | 960 (23.8%) |  |  | 1972 (52.1%) |  |
| Subtotal Gastrectomy | 3082 (76.2%) |  |  | 1815 (47.9%) |  |
| **Surgical access** |  | 0 (0%) |  |  | 0 (0%) |
| Open | 923 (22.8%) |  |  | 2486 (65.9%) |  |
| Laparoscopic or robot-assisted | 3076 (76.1%) |  |  | 1144 (30.2%) |  |
| Converted | 43 (1.1%) |  |  | 157 (4.1%) |  |
| **Lymph node dissection** |  | 0 (0%) |  |  | 0 (0%) |
| D1 | 34 (0.8%) |  |  | 234 (6.2%) |  |
| D1+ | 1923 (47.6%) |  |  | 390 (10.3%) |  |
| D2 | 2024 (50.1%) |  |  | 2749 (72.6%) |  |
| D2+ | 60 (1.5%) |  |  | 369 (9.7%) |  |
| D3 | 1 (0.0%) |  |  | 45 (1.2%) |  |
| **Multivisceral resection**† | 541 (13.4%) | 0 (0%) |  | 912 (24.1%) | 0 (0%) |
| **pT stage** |  | 204 (5.0%) |  |  | 13 (0.3%) |
| pT0 | 57 (1.4%) |  |  | 145 (3.8%) |  |
| pTis | 2123 (52.5%) |  |  | 798 (21.1%) |  |
| pT1 | 494 (12.2%) |  |  | 502 (13.3%) |  |
| pT2 | 644 (15.9%) |  |  | 1230 (32.5%) |  |
| pT3 | 519 (12.8%) |  |  | 1036 (27.4%) |  |
| pT4 | 1 (0.0%) |  |  | 42 (1.1%) |  |
| pTx | 0 (0%) |  |  | 21 (0.6%) |  |
| **pN stage** |  | 420 (10.4%) |  |  | 5 (0.1%) |
| pN0 | 2418 (59.8%) |  |  | 1726 (45.6%) |  |
| pN1 | 462 (11.4%) |  |  | 589 (15.6%) |  |
| pN2 | 366 (9.1%) |  |  | 579 (15.3%) |  |
| pN3 | 375 (9.3%) |  |  | 875 (23.1%) |  |
| pNx | 1 (0.0%) |  |  | 13 (0.3%) |  |
| *SD* Standard deviation *BMI* Body Mass Index *ASA* American Society of Anesthesiologists | | | | |  |
| † Additional resection of adjacent organ(s), including cholecystectomy. | | | | | |

| **Supplementary Table 3** Postoperative complications and adverse outcomes, stratified per region. |  |  |  |  |  |
| --- | --- | --- | --- | --- | --- |
| *Variables* | *East Asia (n = 4042)* | |  | *Europe/America (n = 3787)* | |
|  | **No. (%)** | **Missing (%)** | **No. (%)** | | **Missing (%)** |
| **Postoperative complications** |  |  |  |  |  |
| Pulmonary complications* | 104 (2.6%) | 0 (0%) |  | 332 (8.8%) | 0 (0%) |
| Anastomotic leakage † | 108 (2.7%) | 0 (0%) |  | 255 (6.7%) | 0 (0%) |
| Intra-abdominal abscess ‡ | 187 (4.6%) | 0 (0%) |  | 114 (3.0%) | 0 (0%) |
| Ileus/motility disorder § | 75 (1.9%) | 0 (0%) |  | 108 (2.9%) | 0 (0%) |
| Fistula (pancreatic and/or lymphatic) | 61 (1.5%) | 0 (0%) |  | 127 (3.4%) | 0 (0%) |
| Surgical site infection | 67 (1.7%) | 0 (0%) |  | 55 (1.5%) | 0 (0%) |
| Cardiac ¶ | 17 (0.4%) | 0 (0%) |  | 80 (2.1%) | 0 (0%) |
| Intra-abdominal bleeding | 20 (0.5%) | 0 (0%) |  | 71 (1.9%) | 0 (0%) |
| Stenosis of anastomosis (with clinical symptoms) | 71 (1.8%) | 0 (0%) |  | 14 (0.4%) | 0 (0%) |
| Luminal bleeding | 21 (0.5%) | 0 (0%) |  | 41 (1.1%) | 0 (0%) |
| Ischemia ˚ | 39 (0.5%) | 0 (0%) |  | 30 (0.8%) | 0 (0%) |
| Renal insufficiency • | 6 (0.1%) | 0 (0%) |  | 24 (0.6%) | 0 (0%) |
| Other ∞ | 111 (2.7%) | 0 (0%) |  | 308 (8.1%) | 0 (0%) |
| Total proportion of patients with complication | 737 (18.2%) | 0 (0%) |  | 1147 (30.3%) | 0 (0%) |
| **Adverse outcomes** |  |  |  |  |  |
| Escalation of care ** | 77 (1.9%) | 0 (0%) |  | 255 (6.7%) | 272 (7.2%) |
| Reoperation †† | §§ | 2554 (63.2%) |  | 357 (9.4%) | 0 (0%) |
| Readmission | §§ | 2554 (63.2%) |  | 421 (11.1%) | 274 (7.2%) |
| Mortality (30-day) | 3 (0.1%) | 0 (0%) |  | 91 (2.4%) | 0 (0%) |
| Prolonged hospital stay ‡‡ | 1104 (27.3%) | 206 (5.1%) |  | 915 (24.2%) | 52 (1.4%) |
| * Pneumonia, pleural effusion, respiratory failure, pneumothorax and/or pulmonary embolism. | | | | | |
| † Any clinically or radiologically proven anastomotic leakage. | |  |  |  |  |
| ‡ Intra-abdominal abscess and/or abdominal collections without anastomotic leak. | | | | |  |
| § Postoperative paralytic ileus, mechanical ileus or delayed gastric emptying. | | | | |  |
| ¶ Supraventricular and ventricular arrhythmia, myocardial infarction and/or heart failure. | | | | |  |
| ˚ Postoperative bowel perforation or necrosis. |  |  |  |  |  |
| • Acute renal failure, need for dialysis. |  |  |  |  |  |
| ∞ Includes: vascular-, urinary-, gastro-intestinal complications, hepatic insufficiency, cholecystitis and other type of infections.  ** Unplanned readmission to a unit of higher surveillance (either intermediate- or intensive care unit).  †† Surgical intervention under general anesthesia.  ‡‡ >75th percentile, stratified for surgical access. | | | | | |
| §§ More than 60% missing. |  |  |  |  |  |
|  |  |  |  |  |  |
|  |  |  |  |  |  |

| **Supplementary Table 4** Population attributable fractions of escalation of care and complications after gastrectomy, stratified per region. | | | | | | | |
| --- | --- | --- | --- | --- | --- | --- | --- |
| **Postoperative complication** | *East Asia (4042)* | | |  | *Europe/America (n = 3515)* | | |
|  |  |  |  |  |  |  |  |
|  | **No. with/without escalation of care** | **Adjusted PAF (95% CI)** | ***p*-value** |  | **No. with/without escalation of care** | **Adjusted PAF (95% CI)** | ***p*-value** |
|  |  |  |  |  |  |  |  |
| Pulmonary complications | 27/77 | 28.4 (16.2 - 40.5) | < 0.001 |  | 90/221 | 14.2 (6.5 - 21.9) | < 0.001 |
| Anastomotic leakage | 22/86 | 21.4 ( 10.5 - 32.3) | < 0.001 |  | 99/128 | 27.4 (20.8 - 34.0) | < 0.001 |
| Abdominal collection | 7/180 | 4.3 (-0.3 - 11.8) | 0.260 |  | 12/94 | 3.1 (0.3 - 5.9) | 0.028 |
| Ileus/motility disorder | - | - | - |  | - | - | - |
| Fistula (pancreatic and lymphatic) | - | - | - |  | - | - | - |
| Surgical site infection | - | - | - |  | - | - | - |
| Cardiac | 7/10 | 5.6 (0.0 - 11.3) | 0.056 |  | 34/42 | 8.4 (4.3 - 12.6) | < 0.001 |
| Intra-abdominal bleeding | 12/8 | 13.6 (5.8 - 21.3) | < 0.001 |  | 24/41 | 3.4 (0.3 - 6.5) | 0.034 |
| Stenosis of anastomosis | - | - | - |  | - | - | - |
| Luminal bleeding | 6/15 | 5.7 (0.5 - 11.1) | 0.033 |  | 13/25 | 2.2 (0 - 4.5) | 0.059 |
| Ischemia | 1/8 | 1.2 (-0.1 - 3.8) | 0.386 |  | 13/15 | 3.2 ( 0.8 - 5.7) | 0.010 |
| Renal insufficiency | - | - | - |  | - | - | - |
| *PAF* Population attributable fraction *CI* Confidence interval | | | | | | |  |

| **Supplementary Table 5** Population attributable fractions of complications on prolonged hospital stay after gastrectomy, stratified per region. | | | | | | | |
| --- | --- | --- | --- | --- | --- | --- | --- |
| **Postoperative complication** | *East Asia (4042)* | | |  | *Europe/America (n = 3735)* | | |
|  |  |  |  |  |  |  |  |
|  | **No. with/without prolonged hospital stay** | **Adjusted PAF (95% CI)** | ***p*-value** |  | **No. with/without prolonged hospital stay** | **Adjusted PAF (95% CI)** | ***p*-value** |
|  |  |  |  |  |  |  |  |
| Pulmonary complications | 68/18 | 2.2 (1.3 - 3.1) | < 0.001 |  | 195/126 | 6.2 (3.9 - 8.4) | < 0.001 |
| Anastomotic leakage | 83/13 | 3.7 (2.6 - 4.8) | < 0.001 |  | 207/36 | 12.2 (10.0 - 14.3) | < 0.001 |
| Abdominal collection | 155/20 | 8.4 (6.9 - 9.9) | < 0.001 |  | 90/24 | 4.8 (3.4 - 6.1) | < 0.001 |
| Ileus/motility disorder | 57/14 | 2.8 (1.9 - 3.7) | < 0.001 |  | 74/34 | 3.4 (2.2 - 4.7) | < 0.001 |
| Fistula (pancreatic and lymphatic) | 39/18 | 1.6 (0.8 - 2.3) | < 0.001 |  | 84/42 | 3.8 (2.4 - 5.1) | < 0.001 |
| Surgical site infection | 45/19 | 1.3 (0.6 - 2.1) | < 0.001 |  | 27/28 | 0.8 (0.0 - 1.6) | 0.077 |
| Cardiac | 10/4 | - | - |  | 35/39 | - | - |
| Intra-abdominal bleeding | 17/2 | 0.6 (0.2 - 1.1) | 0.011 |  | 42/28 | 0.7 (-0.2 - 1.6) | 0.157 |
| Stenosis of anastomosis | 49/15 | 2.2 (1.3 - 3.0) | < 0.001 |  | 9/5 | 0.6 (0.0 - 1.1) | 0.037 |
| Luminal bleeding | 13/6 | 0.4 (0.0 - 0.8) | 0.070 |  | 27/14 | 1.1 (0.04 - 1.8) | 0.002 |
| Ischemia | 9/0 | 0.2 (0.0 - 0.4) | 0.043 |  | 19/10 | 0.8 (0.1 - 1.5) | 0.029 |
| Renal insufficiency | 4/2 | - | - |  | 14/9 | - | - |
| *PAF* Population attributable fraction *CI* Confidence interval | | | | | | | |

| **Supplementary Table 6** Population attributable fractions of 30-day mortality and complications after gastrectomy, stratified per region. | | | |
| --- | --- | --- | --- |
| **Postoperative complication** | *Europe/America (n = 3787)* | | |
|  |  |  |  |
|  | **No. with/without mortality** | **Adjusted PAF (95% CI)** | ***p*-value** |
|  |  |  |  |
| Pulmonary complications | 32/300 | 24.1 (3.1 - 45.0) | 0.024 |
| Anastomotic leakage | 35/220 | 27.6 (15.2 - 39.9) | < 0.001 |
| Abdominal collection | - | - | - |
| Ileus/motility disorder | - | - | - |
| Fistula (pancreatic and lymphatic) | - | - | - |
| Surgical site infection | - | - | - |
| Cardiac | 21/59 | 22.5 (4.1 - 40.9) | 0.017 |
| Intra-abdominal bleeding | - | - | - |
| Stenosis of anastomosis | - | - | - |
| Luminal bleeding | - | - | - |
| Ischemia | 11/19 | 19.2 (-5.9 - 44.4) | 0.133 |
| Renal insufficiency | 7/17 | 16.5 (-14.9 - 47.9) | 0.303 |
| *PAF* Population attributable fraction *CI* Confidence interval | | | |

| **Supplementary Table 7** Population attributable fractions of reoperation and complications after gastrectomy, stratified per region. | | | |
| --- | --- | --- | --- |
| **Postoperative complication** | *Europe/America (n = 3787)* | | |
|  |  |  |  |
|  | **No. with/without escalation of care** | **Adjusted PAF (95% CI)** | ***p*-value** |
|  |  |  |  |
| Pulmonary complications | - | - | - |
| Anastomotic leakage | 143/112 | 31.4 (25.9 - 36.8) | < 0.001 |
| Abdominal collection | - | - | - |
| Ileus/motility disorder | 35/73 | 4.3 (1.4 - 7.3) | 0.004 |
| Fistula (pancreatic and lymphatic) | 16/116 | 2.8 (0.0 - 5.1) | 0.021 |
| Surgical site infection | - | - | - |
| Cardiac | - | - | - |
| Intra-abdominal bleeding | 27/44 | 8.1 (5.0 - 11.0) | < 0.001 |
| Stenosis of anastomosis | 6/8 | 1.2 (-0.0 - 2.4) | 0.054 |
| Luminal bleeding | 15/26 | 1.9 (0.0 - 3.7) | 0.039 |
| Ischemia | 21/9 | 4.4 (2.3 - 6.5) | < 0.001 |
| Renal insufficiency | - | - | - |
| *PAF* Population attributable fraction *CI* Confidence interval | | | |

| **Supplementary Table 8** Population attributable fractions of readmissions and complications after gastrectomy, stratified per region. | | | |
| --- | --- | --- | --- |
| **Postoperative complication** | *Europe/America (n = 3513)* | | |
|  |  |  |  |
|  | **No. with/without escalation of care** | **Adjusted PAF (95% CI)** | ***p*-value** |
|  |  |  |  |
| Pulmonary complications | - | - | - |
| Anastomotic leakage | 52/175 | 3.8 (0.3 - 7.2) | 0.030 |
| Abdominal collection | 29/77 | 3.0 (0.8 - 5.3) | 0.009 |
| Ileus/motility disorder | 29/74 | 3.2 (0.9 - 5.5) | 0.005 |
| Fistula (pancreatic and lymphatic) | - | - | - |
| Surgical site infection | 15/31 | 1.9 (0.4 - 3.5) | 0.013 |
| Cardiac | 9/67 | 1.7 (0.0 - 3.3) | 0.043 |
| Intra-abdominal bleeding | - | - | - |
| Stenosis of anastomosis | - | - | - |
| Luminal bleeding | - | - | - |
| Ischemia | - | - | - |
| Renal insufficiency | - | - | - |
| *PAF* Population attributable fraction *CI* Confidence interval | | | |

**Supplementary figure 1** Spread of number of complications for patients with more than 1 complication.

**
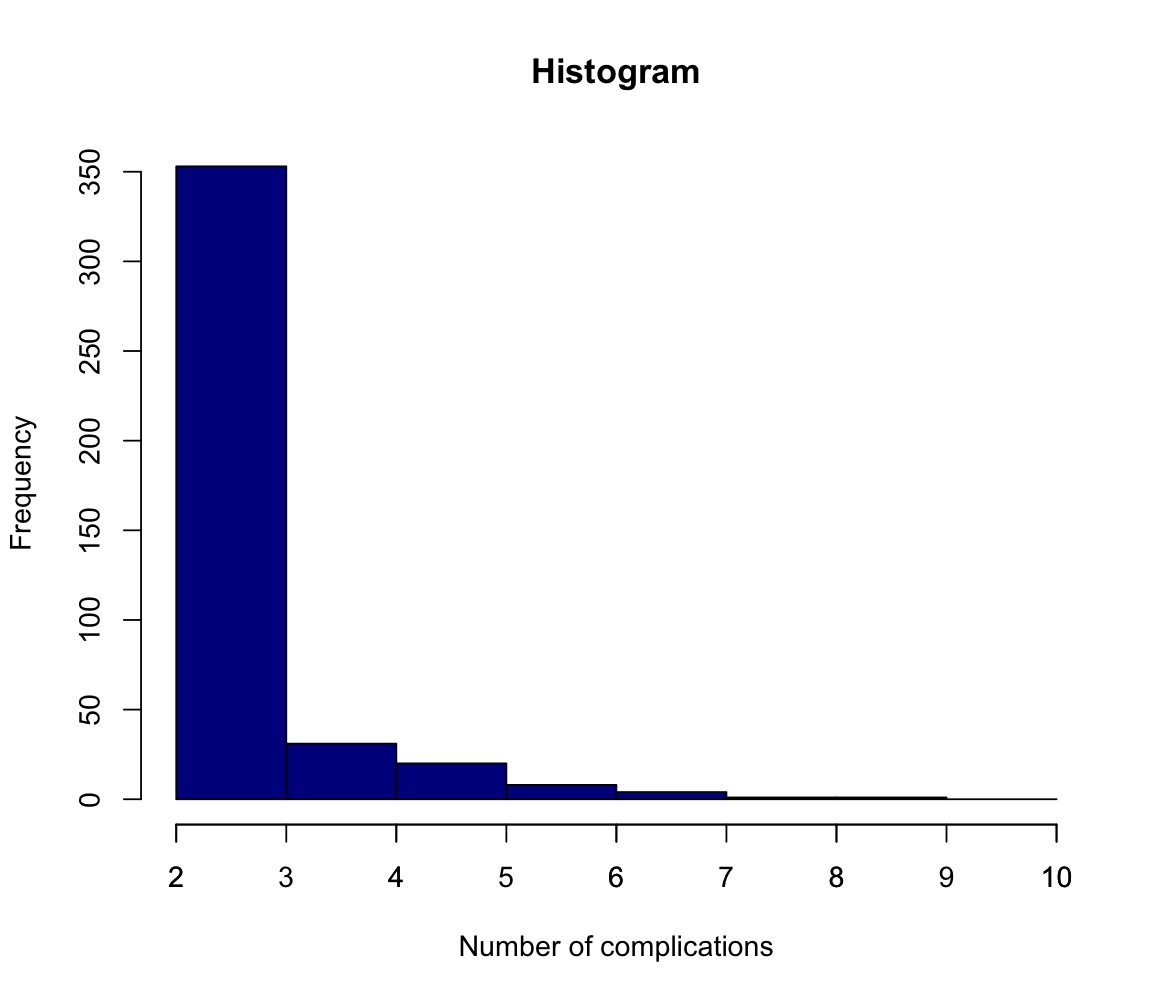
**

**Supplementary Figure 2.** Study flowchart


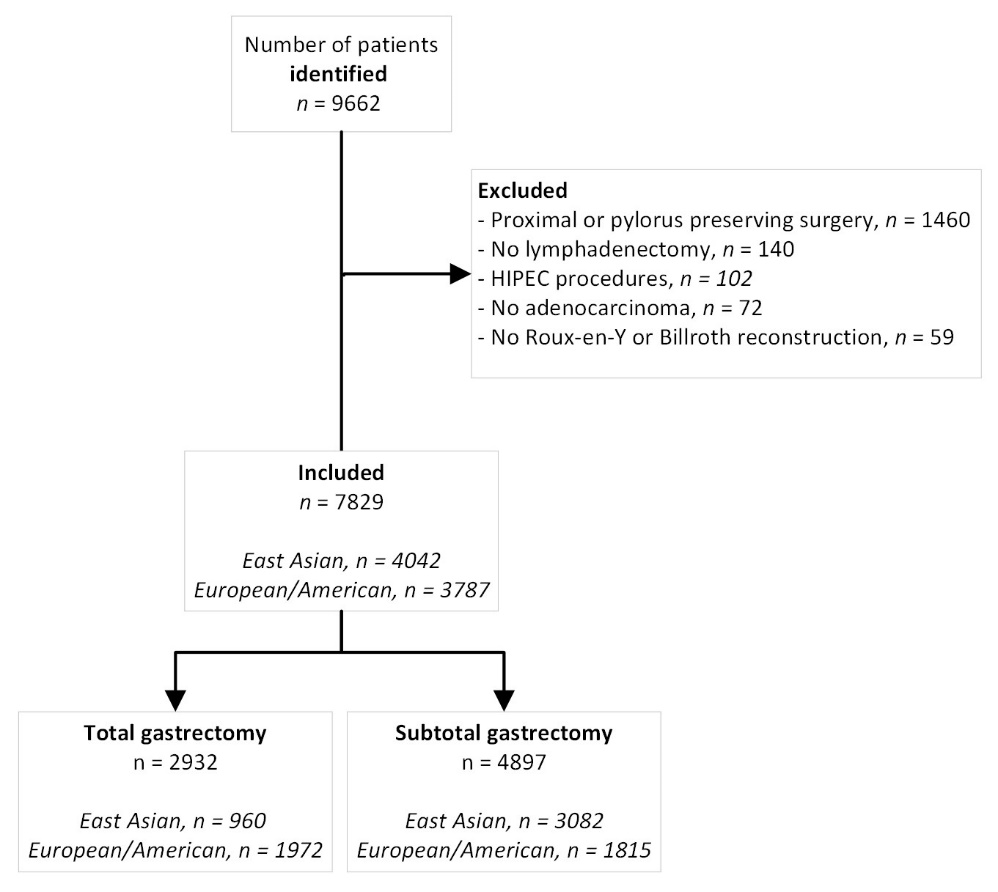


**Supplementary figure 3** A correlation plot visualizing the correlation between complications and adverse clinical outcomes. Spearman’s correlation coefficient was used to assess correlation. Each cell represents the correlation coefficient between two variables, with the colour indicating the strength and direction of their correlation (ranging from -1 to 1).


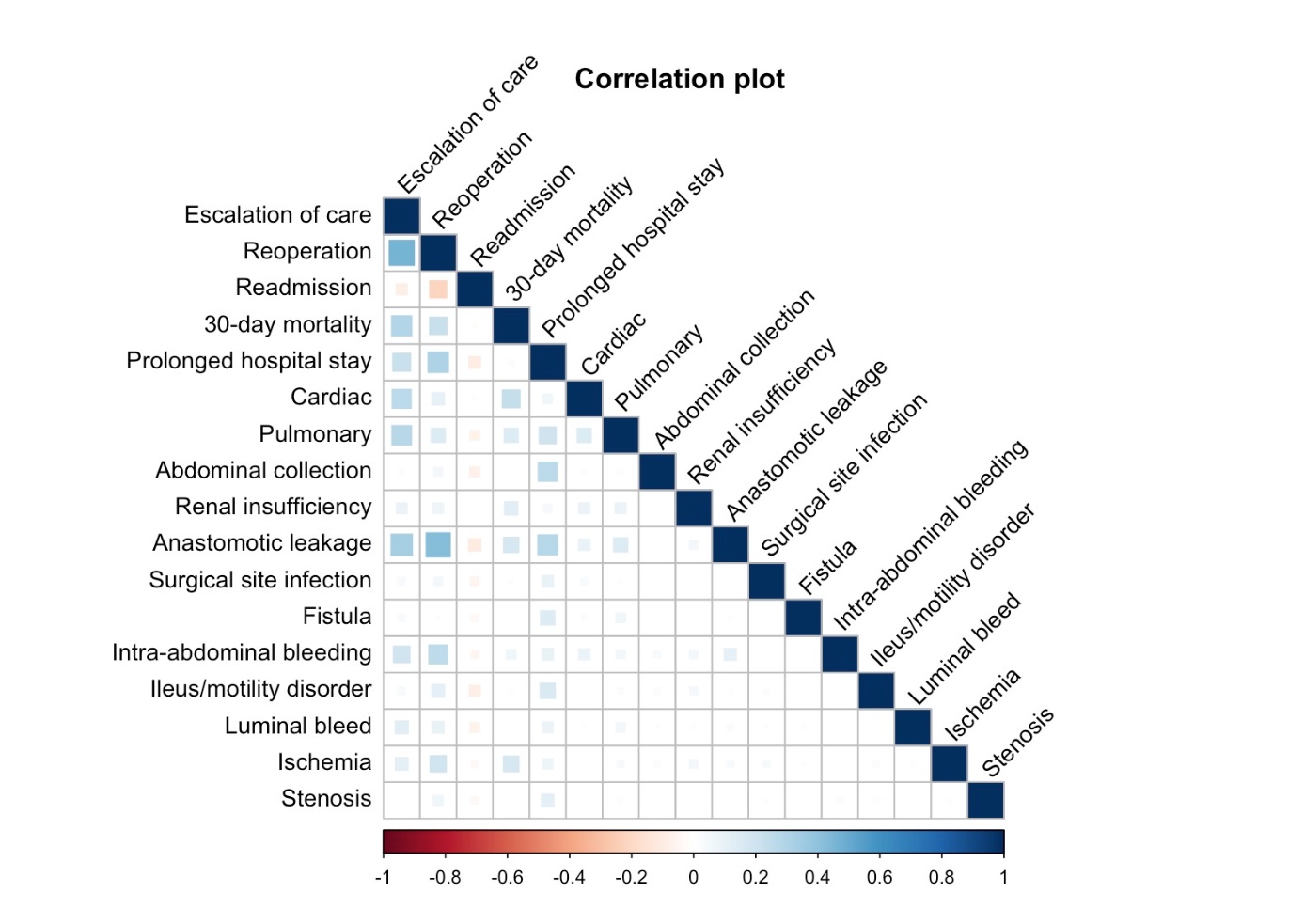

Supplement: znaf043_Supplementary_Data [file znaf043_supplementary_data.docx]
